# The rationale of ethanol inhalation for disinfection of the respiratory tract in SARS-CoV-2-positive asymptomatic subjects

**DOI:** 10.11604/pamj.2021.40.201.31211

**Published:** 2021-12-04

**Authors:** Pietro Salvatori

**Affiliations:** 1 Retired Head & Neck Surgeon, Former Head of Ear, Nose and Throat (ENT) Department at the Humanitas San Pio X Hospital, Milan, Italy

**Keywords:** SARS-CoV-2, COVID-19, ethanol, inhalation, public health

## Abstract

Severe Acute Respiratory Syndrome Coronavirus 2 (SARS-CoV-2) pandemic is a major health concern and is affecting the socio-economic lives. As other highly contagious diseases, it is of outmost importance to early identify and treat the healthy carriers or positive asymptomatic subjects (PAS). SARS-CoV-2 entry points are mainly in the respiratory tract. No specific virucidal treatments against SARS-CoV-2 are currently available. Monoclonal antibodies are under evaluation, but high cost and possible ineffectiveness against virus variants could limit its use. Resorting nonspecific drugs is an alternative approach. Among them, ethanol (EtOH) is known to be a powerful, cost-effective and abundant virucidal agent, now advised for surgical hand and surfaces disinfection. The paper aims to determine the potential role of inhaled ethanol to disinfect SARS-CoV-2 PAS, taking into account the dimension of the problem, ethanol efficiency and other beneficial effects on the respiratory tract, ethanol local and general toxicity and ethanol therapeutic window; consequently, to propose a study in order to verify this hypothesis. Together with the consolidated knowledge, an extensive review of the medical literature has been carried out looking for sound data able to support (or discard) the rationale on which a study could be built up. Evident data supporting the inhaled ethanol potential role on SARS-CoV-2 PAS disinfection have been found and discussed. A clinical trial to test the hypothesis that inhaled ethanol could be rapidly efficient in lowering or eradicating SARS-CoV-2 from the respiratory tract in PAS is advisable. Individual and public health benefits are stressed, together with socio-economic positive fallouts.

## Perspectives

Severe acute respiratory syndrome coronavirus 2 (SARS-CoV-2) outbreak has hit the global community and we are experiencing the third wave after the first phase - and likely, a fourth and fifth ones - as well as more aggressive variants surge (Delta, Epsilon and Omicron). To date, no specific treatment is definitively recognised as effective. Mass vaccination is expected to significantly improve disease control, but it is time-consuming and raises concerns about efficacy against variants and long-standing protection. In Italy, fully vaccinated subjects could be infected by Delta variant at 12% rate. As a result, the role of prevention over the pandemic control increases, and efforts to understand the transport chain and possible active elimination of the virus become of outmost importance. Actually, individuation and - possibly - treatment of spreading subjects is a main goal to be achieved for the control of any contagious disease. In this view, studies have been carried out on the virus binding receptors as the primary target: conjunctival cells, goblet cells of the upper respiratory tract, type 2 pneumocytes and enterocytes. SARS-CoV-2 entry factors - mostly Angiotensin-converting enzyme 2 (ACE2) receptors - are highly expressed in epithelial cells of the nasal cavity and lower respiratory tract, including alveolar cells (Sungnak 2020) [1] and therefore COVID-19 infection occurs initially in the epithelial layer of the upper respiratory tract, followed by transfer to the lower respiratory tract (Samuel B Polak 2020) [2].

A measure of the effectiveness of containment can be derived from a recent study [3] conducted on the population of Wuhan (around 10,000,000 people), which shows that after containment, the rate of symptomatic positives was lowered to 0.00303%. A large number of these infected subjects do not progress to any clinical form of disease: they are the so-called “asymptomatic positives” (healthy carriers). Positive asymptomatic subjects carry a marked SARS-CoV-2 viral load, thus highlighting their role in the spread of the epidemic. To date there are not certain criteria that allow to individuate the asymptomatic subjects who in turn will infect other subjects, then all of them should be considered suitable to undergo disinfection. Therefore, there is great interest in early identification and possibly treatment of asymptomatic positive subjects. The objective is to interrupt the chain of contagion, to shorten or even eliminate the duration of confinement (with the associated economic, social and emotional costs) and to quickly reintegrate healthy carriers into society. Undeniably, the only option available to asymptomatic positive subjects at present is the 14-day quarantine. From Liu *et al*. [4], who studied SARS-CoV-2 contamination in quarantine rooms, it can be inferred that this measure is likely to fail largely, unless the subject lives alone, or each member of the household have their own bedroom, kitchen and bathroom etc.

The purpose of the present paper is to illustrate the dimension of the problem, to depict the current options, to examine the elements of efficacy and toxicology which may justify the use of inhaled ethanol (or ethyl alcohol) for the disinfection of the airways, in subjects contaminated by SARS-CoV-2 and without symptoms.

**Methods:** in this paper, we searched databases, including MEDLINE, Embase, Europe PubMed Central, medRxiv, and bioRxiv, and the grey literature, for research articles published up to 29^th^ July, 2021. We included case series (with five or more participants), cohort studies, randomised controlled trials and databases of trials registration dealing with: i) epidemiological data illustrating the dimension of the problem; ii) current efforts to disinfect/clear SARS-CoV-2 asymptomatic positive subjects; iii) the power of ethanol to destroy - or inactivate - viruses in general and SARS-CoV-2 in particular; iv) ethanol potential beneficial actions on the airways; v) local and general toxic effects of ethanol, either ingested or inhaled; vi) data allowing the outline of the therapeutic window of inhaled ethanol. Sound data were considered in order to support (or discard) the rationale of the proposed novel approach.

**Dimension of the problem:** currently 29^th^ July 2021, the world active cases are 196,717,438 and total deaths reached 4,203,776 [5]. The rate of asymptomatic positives (healthy carriers) ranges 17 to 20% [6]. Asymptomatic positive subjects become symptomatic (to any degree) at the rate of 43%, within 8 days (mean) [7]. As many countries adopt some quarantine programs, repercussions on social and economic fields are hugely negative. According to a very recent meta-analysis, the mean viral load elimination time is 14 days for the lower respiratory tract and 17 days for the upper respiratory tract (Cevik) [8]. Interestingly, no viable virus has ever been detected 9 days after the onset of the disease. Comparison between asymptomatic and symptomatic patients produced conflicting results between the two groups regarding the elimination time.

**Current efforts:** i) inhaled administration (as a solution of pure ethanol) of ivermectin, an antiparasitic medicine with antiviral properties, is currently being studied; ii) monoclonal and polyclonal antibodies, highly specific drugs, are currently under evaluation and use, but the potential benefits are seriously limited by their high cost and the possible loss of efficacy due to variants; iii) in the absence of specific proven treatments for respiratory disinfection, efforts are warranted to explore the potential of nonspecific drugs as well. Attempts to disinfect positive asymptomatic subjects have been made by Guenezan *et al*. [9]. In one small randomized clinical trial, povidone iodine nasal spray and gargle mouthwash resulted in significant reduction of viral titer, but had no effect on the lower respiratory tract; iv) drugs enhancing ACE2 activity are under evaluation.

**Ethanol efficiency:** certainly, the use of ethyl alcohol, or ethanol, is omnipresent in the practice of disinfection. In addition, there is a large amount of consolidated data that demonstrates the antiviral action of ethanol, possibly due to the action of the solvent on lipids (pericapsid) and denaturation of proteins (capsid) [10]. This effect depends on the temperature and the phase in which the pericapsid is located (which derives from the cell membrane of the infected host). Using an aqueous solution of 35.2% by weight (equal to 44% by volume) ethanol, the effect is maximised at around 50°C (crystalline phase) and minimised or ineffective at around 25°C (gel phase). At human body temperature, it is reasonable to estimate an intermediate effect. Ethanol has been shown to have a direct impact on human coronaviruses, such as Severe Acute Respiratory Syndrome Coronavirus (SARS), Middle East Respiratory Syndrome (MERS), Human Endemic Coronavirus (HcoV). These viruses can survive for days on surfaces such as plastic and glass. Disinfectants have been shown to reduce the infectivity of the coronavirus in a very short time (<60 seconds), including Ethanol (EtOH) which is 62% to 71% effective. Fortunately, SARS-CoV-2 is an enveloped virus that is very sensitive to ethanol, and existing experimental data indicates that an ethanol concentration of 30% v/v is sufficient to inactivate SARS-CoV-2 in 30 seconds (Kratzel, 2020) [11]. Manning *et al*. [12] calculated the amount of alcohol needed to clear SARS-CoV-2 viral load affecting the lungs. a) the viral load of COVID-19 is estimated at 20 million per mL of lung tissue (20 * 10^6^per mL); b) in 6 * 10^3^mL of lung tissue (adults), there are 120 * 10^9^(billions) of virus particles (rounded to 200 * 10^9^(billions), many of which are infected cells; c) it is assumed that 10 * 10^6^million molecules of ethanol are needed to disinfect or inactivate a viral particle; d) the density of ethanol is approximately 0.8 g/ml = 800 g/l = 800,000 mg/l = 80,000 mg/dl = 800 mg/ml. Its molar mass is 46 g/moI. It should be remembered that a mole content N = 6.02252 * 10^23^(= Avogadro's number) molecules; e) to remove 200 * 10^9^(billions) of viruses, (10 * 10^6^) * (200* 10^9^) = 2* 10^18^molecules of ethanol will be needed (molar mass = 46 g/mol); f) (2* 10^18^EtOH)/(N * 10^23^EtOH/mol) = 3.3 * 10^-6^moles of ethanol; g) (3.3 * 10^-6^) * (46 g/mol) = 0.000153 gr = 153 µg of ethanol or 191.25 µL.

**Ethanol effects on respiratory cells and microbiota:** i) The effect of alcohol on respiratory hairy cells is a bimodal function of both exposure time and dose. Sisson [13] has shown in vitro that brief exposure (10 minutes) of respiratory hair cells to ethanol (10 mM concentration = 0.46 mg/ml) causes a 40% increase in beat frequency (6 Hz to 8.5 Hz). This effect is mediated by a nitrogen oxide - dependent mechanism. Conversely, the same experiment carried out with ethanol at a higher concentration (1 M = 46 mg/ml) reduced the beat frequency, thus suggesting a toxic effect of ethanol which, by desensitisation, renders stimulation-resistant ciliary motility (a process known as Alcohol-Induced Ciliary Dysfunction mediated by oxidative stress). ii) Until the 1950s, inhaled EtOH was shown to be both effective and safe in the treatment of pulmonary edema and cough treatment. iii) Ethanol is a common excipient in inhalation therapy for asthma and chronic obstructive pulmonary disease, up to 9 mg per actuation. There may be legitimate concerns about the negative impact of EtOH on the respiratory microbiota, but the medical literature lacks direct data on this subject. On the contrary, some positive suggestions could be derived. Indeed, in a set of patients intubated with COVID-19, Sulaiman *et al*. [14] found that a poor clinical result was associated with an enrichment of the microbiota of the lower respiratory tract with an oral commensal (*Mycoplasma salivarium*) and a viral load elevated SARS-CoV-2. Rueca *et al*. [15] studied the nasal/oropharyngeal microbial flora and observed complete depletion of Bifidobacterium and Clostridium exclusively in intensive care patients due to SARS-CoV-2.

**Ethanol toxicity:** from a toxicological point of view, there is a substantial difference between ingested ethanol and inhaled ethanol: the latter directly reaches the left ventricle of the heart and then the brain, thus skipping the first obligatory metabolic step of ingested ethanol. Primarily, there are four real-world models in which the toxicity of acute inhalation of ethanol has been (or is) studied.

**1) Surgical disinfection of the hands:** Bessonneau [16] has shown that during surgical disinfection of the hands with a gel containing ethanol at a concentration of 700 g/l, the cumulative dose of inhaled ethanol in 90s is 328.9mg. Since the inhalation/absorption rate (i.e. the amount of ethanol that passes from the alveoli to the bloodstream) is 62%, the blood alcohol level would be 203.9mg , which gives a blood alcohol level (BAC) of 40.6 mg/L. Hypothetically, even if the absorption of ethanol were instantaneous (not within 90 seconds), the blood alcohol level would be well below the threshold considered toxic (500 mg/L, according to Italian law, and 800 mg/L in most of the United States). Depending on the frequency of surgical hand disinfection associated with appropriate care activities with a high risk of contamination (eg, washing incontinent patients), a healthcare worker may disinfect their hands up to 30 times per day [17] resulting in a daily dose of inhaled ethanol of 9.86 grams.

**2) The liquids used in some “electronic cigarette”:** smoke contain ethanol in various proportions. More [18] reports ethanol absorption data related to the use of electronic cigarettes containing 23.5% ethanol, used with different suction models. In no case did the estimated blood alcohol level exceed 0.85 mg/l. By extrapolating to triple or quadruple concentrations (23.5% x 3 = 70.5%), 23.5% x 4 = 94%, respectively), the expected blood alcohol level should be 0.85 mg/lx 3 = 2.55 mg/L in the first hypothesis and 3.4 mg/L in the second, which are well below the toxic threshold.

**3) COVID-19-pneumonia:** patients are currently being evaluated for treatment with ethanol inhalation [19].

**4) A phase II clinical trial:** to evaluate the efficacy and safety of inhaled ethanol in the treatment of COVID-19 at an early stage has also been registered. At present, the trial is actively recruiting patients [20,21]. i) Mucosal or structural damages to EtOH in the lung, trachea and esophagus have been studied by Castro-Balado *et al*. [21] in rodents inhaling 65% v/v ethanol for 15 min every 8 hours (3 times a day), for five consecutive days (flow rate: 2L/ minute) with a calculated absorbed dose of 1.2 g/kg/day. In humans, under the same circumstances, this dose would correspond to 151g/day. In particular, the histological samples revealed no damage, both in treated animals and in controls. ii) Considering the toxicity of chronic ethanol inhalation, numerous studies indicate that industrial exposure is not a risk in reproductive medicine (Irvine) [22] or in oncology (Bevan) [23]. The latter studied the inhalation exposure to the occupational exposure limit (OEL) for the United Kingdom (1000 ppm of ethanol = 1910 mg/m^3^, over an 8-hour shift) and estimated an equivalence of ingestion of 10 g of ethanol (approximately 1 glass of alcohol) per day. These figures strongly agree with those reported by Bessonneau [16] and Boyce [17]. iii) Chronic ethanol use is not the same as chronic ethanol abuse, which can induce lung damage (alveolar macrophage dysfunction, increased susceptibility to bacterial pneumonia and tuberculosis). iv) Given that the blood volume is approximately 5L and the maximum allowable blood level of ethanol is 500 mg/L, it can be stated that in a healthy adult the maximum dose of ethanol that can be administered instantly is 2.5g.

The rate of ethanol elimination varies from 120 to 300 mg/L/hour [24]. Ninety-five percent of ingested (or inhaled) EtOH is metabolised by alcohol dehydrogenase, while the remaining 5% is eliminated - unmodified - by exhaled air, urine, sweat, saliva and tears.

**Inhaled ethanol therapeutic window:** no targeted studies on this topic were found. However, data available from regulatory reports will help to set the maximum allowed ethanol dose or concentration [23]. Each type of inhalation therapy for airway diseases is potentially more effective than any other form of administration [12].

**Dimension of the problem:** the pattern of SARS-CoV-2 outbreak shows a quite constant progression mixed with local upsurges, probably due to variants selection and superspreader events [25]. Besides the priceless value of lost lives (4,203,776 so far)and suffering endured, the world lost economic output has reached the tremendous level of almost 3.94 trillion U.S.dollars [26]. Reasonably, these data justify the extensive treatment of positive asymptomatic subjects in order to slow down or, hopefully, block the contagion.

**Current efforts:** the study on ivermectin is still ongoing. At present, no study on routine monoclonal antibodies treatment of SARS-CoV-2 positive asymptomatic subjects has been published yet. Moreover, the potential benefits appears to be seriously limited by their high cost and the possible loss of efficacy due to variants. Povidone iodine [9] has showed great effectiveness on reducing the viral titre on pharynx and oral cavity. However, the lower respiratory tract is not reached by povidone iodine gargles and this poses a remarkable limitation. Nevertheless, this work deserves special attention, as it focuses on the treatment of a fundamental step in the chain of viral transmission. Of course, ethanol inhalation overcomes the above restraint. As regard drugs enhancing ACE2 activity, they are still under evaluation.

**Ethanol efficiency:** experimental and clinical data leave no doubt about ethanol power on destroying or inactivating SARS-CoV-2, even at concentration as low as 30% v/v and short time (30 sec) [11]. Quite probably, ethanol is not effective on the intracellular virus. Considering that viral replication occurs in 48-72 hours - to be followed by cellular death and shedding - it is important to prolong ethanol inhalation at least for 3 days. Moreover, thanks to its non-specificity, ethanol is intrinsically effective on any SARS-CoV-2 variant and other “enveloped” viruses. This feature broadens the ethanol spectrum of action over SARS-CoV-2 pandemic and prospects its use on possible future outbreak caused by such viruses. Theoretical minimal dose of ethanol necessary to eliminate the hypothetical viral load has been calculated (= 153 µg) and results quite low in comparison to daily exposition in many work and voluptuary activities.

**Ethanol effects on respiratory cells and microbiota:** Sisson [13] has shown that the effect of alcohol on respiratory hairy cells is a bimodal function of both exposure time and dose. Ethanol at low concentration (10mm = 0.46 mg/ml) increases ciliary clearance, reasonably contributing to the faster elimination of viral load, which has hopefully been rendered inactive by the physicochemical properties of ethanol itself. Studies about the impact over respiratory microbiota of short-term ethanol administration are lacking. However, some suggestions can be derived on this matter. Indeed, worse outcomes on intesive care unit (ICU) patients were related to the abnormal presence of *Mycoplasma salivarium* into the lower tract or *Clostridia*absence in the upper tract. Interestingly, it should be noted that Mycoplasma and SARS-CoV-2 (Eterpi *et al*.) [27] and SARS-CoV-2 are completely inactivated by ethanol. Moreover, certain strains of *Clostridia* are known to produce endogenous ethanol and this potential has been exploited industrially in ABE fermentation (acronym) to produce acetone, butanol and ethanol [28]. Hypothetically, the absence of nasopharyngeal *Clostridia* could lead to a lack of local ethanol production and therefore reduced/absent inactivation of SARS-CoV-2 at this level, thus allowing the virus to spread to the lower respiratory tract [2].

**Ethanol toxicity:** acute ethanol exposition is subject to the law and varies according to country or state. For general population, the allowed maximum Blood alcohol concentration (BAC) in USA it ranges from 500 to 800mg/L. In work environment also the law regulates the maximum chronic ethanol exposition. For example, the occupational exposure limit (OEL) in United Kingdom is 1000 ppm of ethanol = 1910 mg/m^3^, over an 8-hour shift, and estimated an equivalence of ingestion of 10g of ethanol (approximately 1 glass of alcohol) per day [23]. These figures go largely beyond the theoretical dose required to eliminate the viral load in the respiratory tract. Concerns about the mucosal damage that inhaled ethanol could induce locally have been frequently and strongly raised. The meticulous work from Castro-Balado *et al*. [21] seems to have definitively eliminated these concerns.

**Inhaled ethanol therapeutic window:** no targeted studies on this topic were found, so one must necessarily relate to the current experience [16,23]. Therefore, being the surgical disinfection by 70% ethanol for 90 a daily gesture and universally recommended and practiced, it seems reasonable and logical to assert that the toxic risk of such acute inhalation - that is to say approximately 330 mg - can be considered as negligible [16]. In fact, even assuming this dose was given instantly to a healthy adult, the concentration of ethanol in the air inspired would be 330 mg/5L (airway volume) = 78 mg/L = 0.078 mg/ml. This concentration is both much lower than that experimentally causing alcohol-induced ciliary dysfunction (i.e., 46 mg/ml) [13] and that permitted by law (i.e., 500 mg/L = 0.5 mg/ml). In fact, being the lung and blood volumes roughly the same, similar figures would be obtained for the concentration of ethanol in the blood, well below the legal toxic dose of 500 mg/L. On the other hand, this dose is much higher (a thousand times) than the minimum dose (153 µg) required to inactivate the calculated viral load in the lungs [12]. Each type of inhalation therapy for airway diseases is potentially more effective than any other form of administration [12]. Aerosol therapy makes it possible to lower the dosages, to reach “hidden” areas, to better target specific cells or compartments, etc.: in short, to increase the bioavailability of drugs. The size of the particles generated - classified according to the Aerodynamic Median Mass Diameter (AMMD) - well relates to the site to be treated. For the purpose in the present paper, the AMMD of the aerosol particles should be 5 µm.

By reason of the relative novel approach proposed in this paper, not surprisingly consolidated data in medical literature are scarce. Focus on dimension of the problem showed that disinfection of asymptomatic positives subjects is of utmost importance in term of individual and public health concerns and related economic negative consequences. Currently, efficient and cost-effective solutions for that problem are lacking. The review and updating of knowledge bear witness - within a well-defined framework - to the high efficiency and acceptable toxicity of inhaled ethanol. Therefore, the treatment of SARS-CoV-2 asymptomatic positive subjects with inhaled ethanol is well justified. As already envisaged by Prof. Shintake [29] on March 17^th^, 2020, and Dr. Amoushahi *et al*. [30] on May 25^th^ 2020 - a clinical trial should be conducted to study its efficacy and tolerance in certain specific situations. Actually, the study would be agile, inexpensive, of simple execution.

The authors post the following propositions: first of all, as vaccination seems not avoiding delta variant infection, it has to be made clear that ethanol treatment is not believed alternative to the vaccination, but rather has to be considered synergistic with. Once proven this treatment is effective, the expected benefits on health would include: i) elimination, or at least reduction, of the viral load on the respiratory tract in times significantly shorter than natural times; ii) reduction of the viral pressure on the immune system of the infected subject, in order to slow down the progression to the disease; iii) reduction of the amount of active virus emitted during coughing or sneezing; iv) reduction of the spread of the infection; v)reduction of biological/health damage (lethality, pulmonary fibrosis, psychiatric disorders etc.).

**If the proposed treatment were effective on health, an enormous fallout benefits should be expected:** i) reduction in the economic burden linked to the lowered (if not stopped) work activity (the drop in Gross Domestic Product for the 2020 is close to 10% worldwide) and hospitalisation costs. Savings should be calculated in billions of euros; ii) faster return to normal life (school, work, sports, travel, reduction of measures restricting personal freedom etc.); iii) by virtue of its nonspecific mechanism of action, ethanol is theoretically active regardless of the variant in circulation; iv) moreover, it could be active on other “enveloped” viruses, possible sources of future epidemic outbreaks; v) the slowing down (see, the blocking) of the viral circulation allows to alleviate the pressure on the vaccination campaign; vi) ethanol is largely available and very cost-effective, allowing even countries with limited economic resources to cope with and efficiently manage SARS-CoV-2 epidemic.

## Conclusion

Therefore, Scientists and Public Health Authorities should wisely consider and strongly promote a study on this topic.
